# The influence of existence–relatedness–growth need satisfaction and job burnout of young university teachers: the mediating role of job satisfaction

**DOI:** 10.3389/fpsyg.2023.1205742

**Published:** 2023-08-03

**Authors:** Yang Yang, Que Ling

**Affiliations:** Institute of Education, Xiamen University, Xiamen, China

**Keywords:** ERG theory, need satisfaction, job burnout, job satisfaction, young university teachers

## Abstract

Job burnout among young university teachers is a serious issue that can have negative consequences for their well-being and job performance, as well as students’ learning outcomes. To identify protective factors for job burnout, this study examined the impacts and mechanism of satisfying existence, relatedness, and growth need on job burnout among 968 young university teachers, using the ERG needs theory framework. The results of the regression analysis and mediation analysis showed that ERG needs satisfaction significantly and negatively predicted job burnout, and job satisfaction mediated this relationship. Besides, the total effect of existence need satisfaction on job burnout was significantly smaller than that of relatedness need satisfaction and growth need satisfaction. These findings contribute to our understanding of the antecedents of young university teachers’ job burnout and provide a theoretical basis for practical action to protect them from burnout by enhancing their need satisfaction and job satisfaction.

## 1. Introduction

Job burnout is a typical sign of professional mental health issues, which was first explicitly articulated by clinical psychologist Freudellberger. In 1979, Wilard introduced it into the field of education, at which point attention was drawn to studies on teachers’ job burnout. Maslach et al. (2001) defined burnout as a negative emotional state brought on by ongoing stress, characterizing it by emotional exhaustion, depersonalization, and low sense of accomplishment. This negative emotional state could cause low job engagement, poor performance, a lack of commitment, lower productivity, and ultimately has a detrimental effect on organizational outcomes. According to studies, teachers are more vulnerable to job burnout, especially university teachers (Lackritz, 2004; Schaufeli and Bakker, 2004; Schnall et al., 2009; Watts and Robertson, 2011; Khan et al., 2019). This is because, in contrast to those who work in elementary and secondary schools, university teachers are charged with doing scientific research as well as teaching and educating students, all of which are very important and arduous tasks, bringing a lot of work pressure to them.

Young faculty members are the backbone of universities and are crucial to the cultivation of talent and intellectual innovation. However, young teachers are in an even more stressful situation than older teachers (Vesty et al., 2018). They are in the beginning of their careers, facing a lot of pressure from external factors like the disconnect between social expectations and realistic treatment, the conflict between family and work, and the challenging promotion and retention system, as well as internal factors like a lack of social capital, a sparse academic network, a lack of the ability of self-adjustment and inexperience. According to research, the risk of job burnout is four times higher for teachers with less work experience (Henny et al., 2014). Most scholars agree that job burnout is more prevalent among young teachers and tends to affect them more severely (Vesty et al., 2018; Atmaca et al., 2020). Therefore, it is necessary to pay more attention to job burnout among young university teachers.

Job burnout not only impedes young teachers’ personal, physical, and mental development as well as their professional development, but also negatively impacts the quality of high-level talent training, the growth of research and innovation, and the improvement of national innovation capacity. In order to prevent job burnout, it is essential to figure out the protective factors of it. Regrettably, despite the fact that the topic of burnout has received significant attention from scholars (Stelmokienė et al., 2019; Atmaca et al., 2020; Teles et al., 2020), its protective factors has not received nearly as much attention (Lian et al., 2021). Particularly, there are not enough viewpoints that take into account how teacher needs satisfaction affects burnout from a motivational theory standpoint. Self-Determination Theory (SDT) is one of the widely used motivation theories in the educational context, focusing on students’ learning and how to promote it (Ryan and Deci, 2020; Moè et al., 2022). However, this study focuses more on the issues of young teachers’ professional adaptation and job burnout, which is more congruent with the applied scenarios of the existence-relatedness-growth theory–a theory that primarily focuses on individuals’ needs and motivations in the workplace (Alderfer, 1969). Therefore, in order to shed light on the impact of needs satisfaction on young university teachers’ job burnout and its mechanism, this study employs the ERG theoretical framework as a guide, a sizable sample of data from the Nature global survey, and data analysis techniques like correlation, regression, and the Bootstrap test.

### 1.1. Existence-relatedness-growth theory and young university teachers need

Existence-relatedness-growth (ERG) theory is a development of Maslow’s needs theory, considering survival, relationship and development as the three core needs of individuals (Alderfer, 1969). Specifically, existence need are derived from Maslow’s physiological and security needs, relatedness need from social and esteem needs, and growth need from self-actualization (Saif et al., 2012). Based on the ERG theory, the complex needs of young university teachers who is in the early stages of their academic careers can be simplified into three categories: The first category is existence need, which are the fundamental need for stable material necessities that support daily living (Acquah et al., 2021). The satisfaction of young university teachers’ existence need is mostly represented in their opinions of their pay, perks, job security, and working circumstances. The second category is relatedness need, which includes teachers’ emotional needs, interpersonal relationship needs with their students, colleagues and supervisors (Shikalepo, 2020). The satisfaction of young university teachers’ relatedness need is mostly determined by how they feel about the management structure and the effectiveness of their interpersonal connections. The third one is growth need, which mainly refers to young teachers’ desire for professional development and career development opportunities. Young university teachers growth need is about the pursuit of self-fulfillment. Since the process of teaching and learning is constantly evolving (Mastrokoukou et al., 2022), efficient professional training is necessary for young teachers ability development. Besides, ample research autonomy, reasonable occupational challenges, and sufficient promotion opportunities are also the useful way to meet young university teachers growth need. According to ERG theory, it is not the case that a high-level need becomes motivating only after a low-level need has been fulfilled, but all three types of needs coexist and collectively have an effect on a employees’ attitudes and behavior at work (Luthans, 2005; Patricia and Asoba, 2021).

### 1.2. Existence-relatedness-growth need satisfaction and job burnout

Job burnout is the result of persistently unfavorable work emotions. According to Maslach et al. (2001), job burnout in young university teachers can be described as emotional exhaustion, indifferent attitudes toward students and colleagues, and a lack of accomplishment in both teaching and research work. Recent study have found that the existence need, relatedness need, and growth need of university staff are closely related to job burnout (Tkm Thangal et al., 2021).

Young university teachers, who are still in the early stages of their professions and do not yet have a solid financial foundation, are under a lot of financial strain when they take on family duties including marriage, childrearing, and caregiving for the elderly (Lian et al., 2021). Not being able to get their existence need met through their employment can put those young teachers under great pressure to survive and affect their enthusiasm for teaching and research. Therefore, it is not surprising that teachers cite wages and salaries as their top motivating factors (Shikalepo, 2020). A global survey reveals that postdocs become worn out and progressively lose interest in scientific research due to insufficient income and a feeling of precarity (Woolston, 2020). Inadequate physical conditions at work (e.g., outmoded teaching and office equipment, poor quality school facilities and equipment, etc.) can also have an impact on teachers’ productivity and motivation to educate, which can finally lead to burnout (Akpan, 2013; Salifu and Agbenyega, 2013; Gatsinzi et al., 2014). Thus, we assumed that existence need satisfaction would negatively predict teachers job burnout.

Young teachers are in a crucial stage of their careers as they develop their social capital networks. Unmet relatedness need prevent teachers from realizing their professional identities and prevent them from feeling a sense of belonging at work, which leads to job burnout (Lu et al., 2022). Young university teachers are part of a vast network of interpersonal ties, including collegial, hierarchical, and student-teacher relationships, inside the complicated organizational structure of the institution. Previous study discovered that the administrative system and anxiety communicating with parents were important predictors of teachers’ job burnout (Pressley, 2021). Specifically, a hierarchical organizational management culture can make teachers oppressed (Tsang et al., 2021). In contrast, good interpersonal interactions, peer and superior support, and encouragement support and encouragement from superiors can provide teachers a sense of recognition, boosting their level of personal fulfillment and lowering job burnout (Schaufeli and Bakker, 2004; Wang and Xu, 2004; Vesty et al., 2018). Additionally, a strong teacher-student bond generates uplifting emotions and lessens teachers’ emotional weariness and depersonalization (Milatz et al., 2015). Thus, we assumed that relatedness need satisfaction would negatively predict teachers job burnout.

Another crucial element in motivating young teacher is the development of their professional capacities. That is, their needs for growth. Unmet growth demands prevent teachers from self-actualization, and they are unable to derive satisfaction and value from their work, which causes them to lose interest in moving forward. Specifically, professional autonomy has been demonstrated to be a key preventative measure against job burnout, which means that job burnout may occur in teachers if they feel less in control of their work (Vesty et al., 2018). Meanwhile, lack of promotion prospects leads to a crisis in teachers’ professional mental health, which is an important trigger to job burnout (Padilla and Thompson, 2015). Conversely, reducing teachers’ job burnout can be achieved by offering qualified direction, organized training for their professional development, and a supportive environment for teachers growth (Chen, 2015). Additionally, the challenge of the work can serve as a motivational factor, maintaining teachers’ high levels of commitment and passion for their work (Salifu and Agbenyega, 2013). Thus, we assumed that growth need satisfaction would negatively predict teachers’ job burnout.

### 1.3. The mediating role of job satisfaction

Job satisfaction refers to a positive psychological state that results from an employee’s evaluation of all aspects of their job. Specifically, teacher job satisfaction can be described as the overall feelings about their work, profession, working circumstances (Troesch and Bauer, 2017). The existing research have revealed that teachers job satisfaction is linked to an improvement in their positive work attitudes (Zhai et al., 2013). As many scholars suggested, job satisfaction can act as a motivating factor to make employees happy and satisfied with their work, thus helping employees to build up positive psychological capital to avoid job burnout (Saleem et al., 2010; Capone and Petrillo, 2020). Others hold that job satisfaction itself is a job resource, just like the positive psychological capital, and is excellent buffer against job burnout (Struyven and Vanthournout, 2014).

Additionally, based on Troesch and Bauer (2017), young university teachers job satisfaction can be described as a general emotional feelings and opinions of young teachers about their work, such as teaching, research, and administration work, which are closely related to the fulfillment of teachers’ ERG needs. Scholars found that young university teachers’ job satisfaction has been significantly influenced by factors such as payment, the workplace atmosphere, collegiality, and professional development possibilities (Zhou and Li, 2019; Zhu, 2019). If teachers’ needs are not well met, it may leave them in a stressful situation, which may lower their job satisfaction and finally develop into job burnout syndrome (Leiter et al., 2014). Thus, we assumed that job satisfaction would play a mediating role in the relationship between teachers ERG needs satisfaction and job burnout.

### 1.4. The present study

Job burnout is increasingly prevalent among young university teachers and have negative consequences for the sustainable development of higher education. The purpose of this study is to improve understanding of the proctective factors of job burnout using the ERG needs theory framework and to explore the relationship between Existence-relatedness-growth needs satisfaction and job burnout of young university teachers. According to extant study, three hypotheses were proposed, as follows:

*H1*: Existence need satisfaction would negatively predict teachers job burnout.

*H2*: Relatedness need satisfaction would negatively predict teachers job burnout.

*H3*: Growth need satisfaction would negatively predict teachers job burnout.

*H4*: Job satisfaction would play a mediating role in the relationship between teachers ERG needs satisfaction and job burnout.

**Figure 1:**
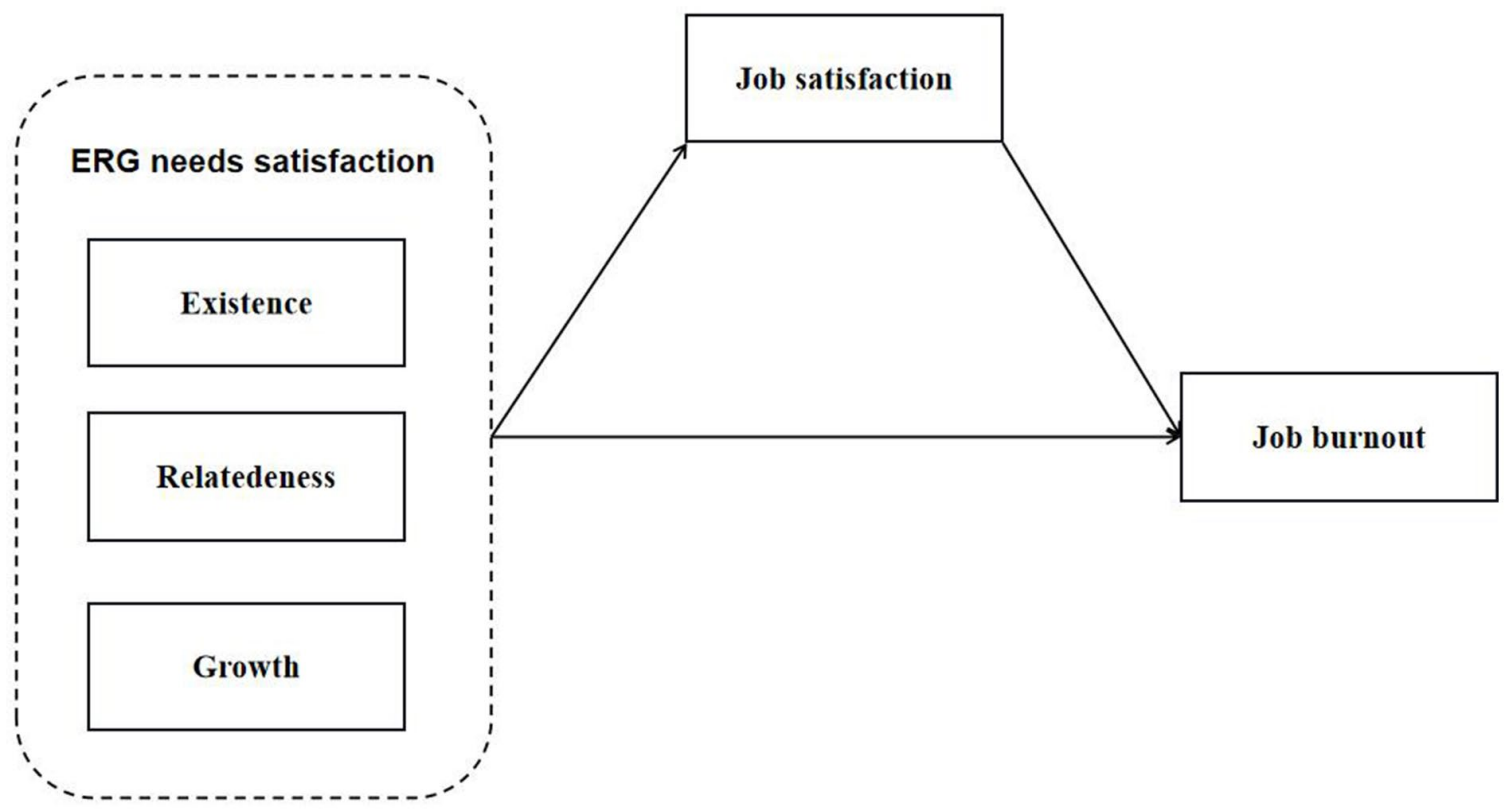
The hypothesized model.

Based on these hypotheses, we outlined a mediating model (see Figure 1) to investigate the association between the ERG needs satisfaction and job burnout in young university teachers as well as the mediating function of job satisfaction. Specifically, in this research model, ERG needs satisfaction is treated as the independent variable, job satisfaction is the mediating variable, and job burnout is the dependent variable.

## 2. Materials and methods

### 2.1. Participants

The data for our research was sourced from the 2021 Nature Salary and Job Satisfaction Survey, which serves as an excellent resource due to its extensive sample size and a the wide range of topics related to working life. On the one hand, the questionnaire was distributed in multiple languages to scientific workers around the world, 3,210 responses from 97 countries were collected. Those respondents came from a wide range of employment sectors including academia, industry and government, and majored in a wide range of disciplines including science, technology and social sciences, which is highly representative. On the other hand, the questionnaire contains a series of questions (e.g., salary and benefits, job satisfaction, job burnout, etc.) to explore the realities of working life (Woolston, 2021a), which is comprehensive inclusion of all the pertinent variables required for our study. However, the survey report solely presented descriptive statistics and concluded that signs of burnout were prevalent (Woolston, 2021b), leaving our research question still unresolved.

The current study places a primary emphasis on young university teachers. By taking into consideration the prevailing conditions and drawing from existing research (Bhatnagar and Das, 2014; Lian et al., 2021), we define “young university teachers” as those aged below 40 years and work in academia. Therefore, only individuals employed in academic fields who were within the age range of 40 years or younger were included in the study’s sample, with a total count of 1,132 participants. In addition, samples with severe missing key variables were excluded, resulting in a valid sample of 968 participants, with an effective sample rate of 85.51%.

### 2.2. Measures

#### 2.2.1. Job burnout

Teachers job burnout was measured in the questionnaire with 13 items including “I feel run down and drained of physical or emotional energy,” “I feel unmotivated and lacking will to complete parts of my job,” etc. The range of values is 1 to 5, with higher values indicating higher levels of job burnout among young university teachers. The mean value of all items was used to reflect teacher job burnout. Cronbach’s α for this scale was 0.885.

#### 2.2.2. Existence-relatedness-growth needs satisfaction

The independent variables include existence need satisfaction, relatedness need satisfaction and growth need satisfaction of young university teachers. Four items in this questionnaire was selected including “Salary/compensation” “Workplace facilities and comfort” was used to measure young teachers existence need satisfaction, five items including “Management and leadership of organization” “Communication with your supervisor” “Relationship with colleagues” was used to measure their relatedness need satisfaction, and four items including “Career advancement opportunities” “Access to workplace-sponsored training and seminars” “Personal sense of accomplishment” to measure their growth need satisfaction. The Cronbach’s α coefficients were 0.723, 0.836 and 0.706, respectively. Besides, confirmatory factor analysis was conducted to assess the validity of those measures. The results of goodness-of-fit indices indicated a reasonably good fit of the measurement model to the data: CMIN/DF = 6.732<8, RMSEA = 0.077<0.8, IFI = 0.906>0.9, CFI = 0.905>0.9, suggesting that the measurement model provides an acceptable fit to the data.

#### 2.2.3. Job satisfaction

The question “How satisfied are you in your current job?” was selected to measure the job satisfaction of young university teachers, with values ranging from 1 to 5. The higher the average score, the higher the degree of teacher job satisfaction.

#### 2.2.4. Control variables

Based on previous studies and the availability of data, job title, gender and worked hours per week were selected as control variables.

### 2.3. Statistical analysis

Data analysis is carried out in the following steps: The first step is data preprocessing. We conduct data cleaning and preliminary organization using Excel and performed reliability analysis, descriptive statistics, and correlation analysis using SPSS21.0, to ensure the feasibility of subsequent data processing. The second step involves conducting direct effect testing. Using Mplus 8.3, a regression model is constructed to examine the total effects of ERG needs satisfaction on job burnout, and the direct effects of ERG needs satisfaction on job burnout and job satisfaction, and job satisfaction on job burnout. The third step involves testing for mediation effects. Through bootstrap analysis with 5,000 resamples, we examine the statistical significance of the mediating effect of job satisfaction between ERG needs satisfaction and job burnout, and we also test the statistical significance of coefficient differences in different pathways using bootstrapping.

## 3. Results

### 3.1. Descriptive statistics and correlations

We analyzed the means, standard deviations, of teachers’ need satisfaction, job satisfaction, and job burnout, and the Pearson correlation coefficients between them. As presented in Table 1, the means indicate that there is still room for improvement in ERG needs satisfaction, job satisfaction and job burnout of young university teachers. Correlation analysis shows that young teachers’ existence need satisfaction, relatedness need satisfaction and growth need satisfaction are significantly positively correlated with their job satisfaction, but negatively correlated with job burnout. Teachers job satisfaction is negatively correlated with their job burnout.

**Table 1:** Descriptives and correlations (*N* = 968).

	*M*	SD	1	2	3	4
1 existence need satisfaction	3.44	0.94	1			
2 relatedness need satisfaction	3.23	0.96	0.49**	1		
3 growth need satisfaction	3.43	0.91	0.54**	0.65**	1	
4 job satisfaction	3.22	1.25	0.52**	0.64**	0.62**	1
5 job burnout	3.01	0.76	−0.36**	−0.53**	−0.48**	−0.53**

** *p* < 0.01; M ranges from 1 to 5.

### 3.2. Total effects and direct effects

The regression model was conducted using ERG needs satisfaction as the independent variables, job satisfaction as the mediating variable, and job burnout as the dependent variable, while gender, job title and worked hours per week were used as control variables. Correlation was allowed between existence need satisfaction, relatedness need satisfaction and growth need satisfaction.

The regression results for the total effect are presented in Table 2. The total effects of teachers’ existence need satisfaction, relatedness need satisfaction and growth need satisfaction on job burnout were − 0.065 [95%CI = (−0.117,-0.011)], −0.273 [95%CI = (−0.334,-0.211)], −0.163 [95%CI = (−0.225,-0.099)], respectively, indicating all of them can significantly and negatively predicted job burnout. Thus, H1 ~ H3 were supported.

**Table 2:** The total effects, indirect effects and pathway differences.

Pathway		Estimate	SE	95% CI	
				Lower	Upper
TOTAL(ENS)		−0.065	0.027	−0.117	−0.011
TOTAL(RNS)		−0.273	0.031	−0.334	−0.211
TOTAL(GNS)		−0.163	0.032	−0.225	−0.099
Indirect1	ENS— > JS— > JB	−0.037	0.008	−0.056	−0.023
Indirect2	RNS— > JS— > JB	−0.067	0.012	−0.093	−0.045
Indirect3	GNS— > JS— > JB	−0.057	0.011	−0.083	−0.038
Diff1	TOTAL(ENS)-TOTAL(RNS)	0.208	0.047	0.115	0.300
Diff2	TOTAL(ENS)-TOTAL(GNS)	0.098	0.045	0.010	0.185
Diff3	TOTAL(RNS)-TOTAL(GNS)	−0.110	0.057	−0.223	0.002

ENS, existence need satisfaction; RNS, relatedness need satisfaction; GNS, growth need satisfaction; JS, job satisfaction; JB, job burnout.

**Figure 2:**
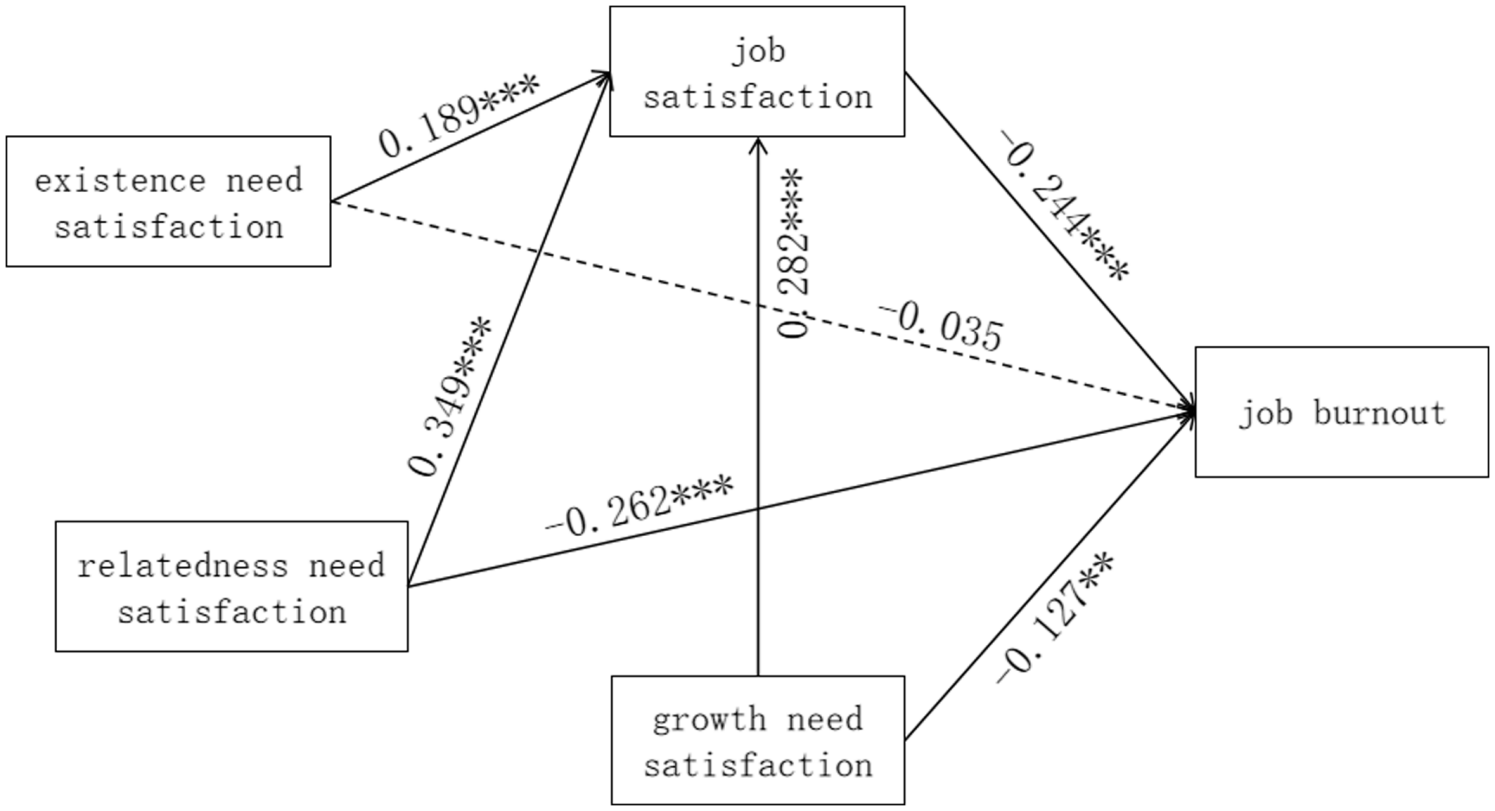
Regression results.

The regression results for the direct effect are presented in Figure 2. The direct effect of teachers’ existence need satisfaction on job burnout was negative but not statistically significant(*β* = −0.035, *p* = 0.285), while the direct effects of relatedness need satisfaction and growth need satisfaction on job burnout were negative and significant(*β* = −0.262, *p* < 0.001; *β* = −0.127, *p* = 0.01). Besides, job satisfaction had a significant negative direct effect on job burnout (*β* = −0.244, *p* < 0.001), and existence need satisfaction, relatedness need satisfaction and growth need satisfaction all had direct effects on job satisfaction (*β* = 0.189, *p* < 0.001; *β* = 0.349, *p* < 0.001; *β* = 0.282, *p* < 0.001).

### 3.3. Mediational effects

The above analysis showed that ERG needs satisfaction and job satisfaction were the key factors influencing job burnout among young university teachers. To further investigate the influence mechanism, a mediation analysis based on bootstrapping (with 5,000 samples) was conducted using Mplus 8.3, to examine the mediation effect of job satisfaction between ERG needs satisfaction and job burnout. The results were shown in Table 2. Existence need satisfaction [*β* = −0.037, SE = 0.008, 95%CI = (−0.056, −0.023)], relatedness need satisfaction [*β* = −0.067, SE = 0.012, 95%CI = (−0.093, −0.045)], and growth need satisfaction [*β* = −0.057, SE = 0.011, 95%CI = (−0.083, −0.038)] indirectly affected job burnout via teachers’ job satisfaction, supporting H4.

In addition, the results of the path difference test showed that the effect of existence need satisfaction on job burnout among young university teachers was significantly weaker than the effect of relatedness need satisfaction[∆ = 0.208, 95%CI = (0.115, 0.300)] and growth need satisfaction [∆ = 0.098, 95%CI = (0.010, 0.185)]. In contrast, there was no significant difference between the effect of relatedness need satisfaction and growth need satisfaction on job burnout [∆ = −0,11, 95%CI = (−0.223, 0.002)].

## 4. Conclusion and discussion

This study utilized the ERG theoretical framework to categorize the needs of young university teachers and investigated the impact of various types of need satisfaction on job burnout using data from a global survey. The findings demonstrated that among young university teachers, satisfaction with existence need, relatedness need, and growth need all negatively predicted job burnout and indirectly influenced job burnout through their effects on job satisfaction. Notably, the total effect of existence need satisfaction on job burnout was found to be significantly weaker than that of relatedness need satisfaction and growth need satisfaction.

In terms of teachers needs satisfaction, previous studies tended to consider a specific type of need individually or treat need satisfaction as a whole, neglecting potential differences in the impact among different types of needs. This study proposed a more comprehensive model to include all types of needs satisfaction using the ERG theoretical framework. Regarding the total effects, our findings indicated that existence need satisfaction negatively associated with young university teachers’ job burnout, supporting the view that a decent wage package could significantly reduce teachers’ stress levels in terms of their basic necessities, thereby enabling them to be more engaged in their work and preventing the job burnout that can occur when they are subjected to ongoing financial hardship (Bashir and Gani, 2020). It is noteworthy that when constructing the regression model with job satisfaction as the mediating variable, the direct impact of existence need satisfaction on job burnout did not pass the significance test, indicating that the protective effect of existence need satisfaction on job burnout primarily occurs through the improvement of job satisfaction. Whether this phenomenon is specific to young teachers or can extend to a broader group of university teachers remains to be explored in future research. Besides, our study revealed a negative correlation between the satisfaction of relatedness need and job burnout. This finding implies that the emotional link between teachers and institution plays a important role in protecting young university teachers from job burnout. Good interpersonal connections help improve young teachers job embeddedness (Lyu and Zhu, 2019) and lessen emotional weariness (Chen and Li, 2020; Tang and Vandenberghe, 2020), which helps them stay enthusiastic and interested in their work and prevents the dominance of negative emotions that can result in crises of occupational mental health. What is more, a negative relationship between growth need satisfaction and job burnout was identified in our study, supporting a positive link between perceived overqualification and job burnout (Chambel et al., 2021). It makes sense that job burnout among university teachers is frequently seen as a result of lacking accomplishment (Fernández-Suárez et al., 2021). Satisfying teachers’ growth need means that they are able to develop professionally in challenging work or advance in their careers, both of which are very beneficial in maintaining and enhancing teachers’ sense of accomplishment, thus effectively reducing job burnout (Padilla and Thompson, 2015; López-Núñez et al., 2020; Oliveira et al., 2021).

This study also found that job satisfaction could be a significant mediator in the association between existence-relatedness-growth needs satisfaction and young teachers’ job burnout. Specifically, ERG needs satisfaction positively predicts job satisfaction among young university teachers, which is supported by expectancy theory (Lawler and Suttle, 1973). Teachers’ needs can be seen as expectations of access to certain material or psychological resources. Having needs met means that teachers are able to fulfill their expectations from their work, which will greatly enhance their motivation and job satisfaction (Ababneh, 2020). Additionally, the significance test was passed by all three types of needs satisfaction, which is a strong endorsement of the ERG hypothesis that those needs are hierarchical but not sequential and that all needs can exist concurrently and affect job satisfaction (Alderfer, 1969; Luthans, 2005; Patricia and Asoba, 2021). On the other hand, job satisfaction negatively predicts young university teachers’ job burnout, which is supported by many scholars (Henny et al., 2014; Troesch and Bauer, 2017; Capone and Petrillo, 2020). This is due to the fact that job satisfaction is in and of itself a positive perception of work, and increased job satisfaction can also lead to young university teachers having more positive emotional experiences (Kumar, 2022). The combination of these multiple positive emotions can create a powerful defense that can protect young teachers from job burnout.

Unexpectedly, we discovered noticeable disparities in the performance of the three types of needs satisfaction in terms of their effects on job satisfaction and job burnout. Specifically, the total effects of relatedness need satisfaction and growth need satisfaction on job burnout exhibited significantly greater strength in comparison to that of relatedness need satisfaction, while there were no significant differences in the impacts of relatedness need satisfaction and growth need satisfaction on job burnout. This can possibly be explained by the characteristics of job burnout. As Wang et al. (2020) suggested, job burnout arises under chronic stress and stress relief is the primary consideration in addressing burnout. On the one hand, the satisfaction of relatedness need suggests that teachers perceive themselves to be in a favorable organizational climate. This climate serves as a protective barrier that enables teachers to establish emotional connections with the university more quickly and to alleviate stress and distress through amicable interpersonal relationships. Therefore, relatedness needs satisfaction can enhance teachers’ psychological capital and energy to cope with stress, ultimately mitigating job burnout. On the other hand, meeting the needs for growth entails the development of teachers’ professional competence, which equips them with the ability to cope with work difficulties and challenges, thereby leading to a reduction in perceived job stress. Besides, job satisfaction, the key mediating role in our model is closely related to the employee’s identification of the value and meaning of the job (Lu et al., 2022). In comparison to existence need, professional and competency growth is more likely to give teachers a sincere appreciation of the value of their work, functioning as a motivator and boosting young teachers’ job satisfaction. Therefore, the enhancement of professional competence and the improvement in job satisfaction may partly explain why meeting the needs for growth has a stronger protective effect against job burnout among young teachers. However, further testing will need to be done in subsequent studies to determine whether this variation in influence is caused by the traits of young teachers who are new to the workforce.

To sum up, existence-relatedness-growth needs satisfaction plays an important role in protect young teachers from job burnout, while job satisfaction a substantial bridge between the two. Accordingly, strengthening administration of the university teaching force in order to better meet teachers’ ERG needs and increase their job satisfaction is a reasonable direction that can be tried to combat young teachers’ job burnout.

## 5. Practical implications

To sum up, existence-relatedness-growth needs satisfaction plays an important role in protect young teachers from job burnout, while job satisfaction a substantial bridge between the two. Accordingly, there are some practical implications for improving organizational conditions and safeguarding young university teachers from experiencing job burnout.

First, providing a decent wage package is an essential foundational matter that universities must address. Young university teachers who have just begun their careers confront significant economic pressures due to the obligations of marriage, parenthood, and caring for elderly family members, even when they have not yet accumulated substantial economic assets (Lian et al., 2021). The salary provided by the university serves as the primary source for them to tackle these economic pressures. Therefore, universities should be attentive to the economic pressures faced by young teachers and develop appropriate compensation systems to aid their adjustment during this transitional period. This would enhance the life satisfaction and well-being of young teachers, enabling them to engage more effectively in teaching and research, while reducing the risk of job burnout. Specifically, universities can address the basic existence needs of young faculty members by focusing on optimizing salary structures, designing performance-based incentive strategies, and implementing other specific measures.

Second, university management has to be aware of the importance of meeting relatedness need in young faculty development. As indicated by our research findings, the fulfillment of relatedness need shows the greatest protective effect against job burnout. Young teachers who have excellent interpersonal interactions are better able to get emotional support from institution, which is benefit for them to maintain a high level of psychological well-being and defense against the occupational mental health problems like burnout. Therefore, efforts should be directed toward cultivating an organizational atmosphere with humanistic care, helping young teachers integrate into the institution community as soon as possible, and assisting them in establishing a positive emotional connection with the organization. Specifically, universities can organize regular faculty social events to provide opportunities and platforms for teachers to network and communicate outside of their work responsibilities, thereby assisting young teachers in developing friendships in their new environment. Additionally, administrators should also place a high priority on offering young teachers with heartwarming care, such as engaging in regular one-on-one communication to inquire about their challenges in work and daily life, providing professional mental health services.

Third, university should also consider the growth need of young teachers from multiple perspectives and establish appropriate paths for their professional development and career advancement. Young teachers are in the early stages of their career development, filled with anticipation and hope for the future. The satisfaction of their growth need contributes to the maintenance of their work enthusiasm and protects them from job burnout. Specifically, it is necessary to establish a fair and transparent promotion mechanism and familiarize young teachers with the rules and requirements for promotion, which are useful to help young teachers set clear career development goals and encourage them to continuously strive toward their goals. Besides, providing challenging tasks and giving young teachers sufficient professional autonomy and research independence can facilitate the full development of their abilities. Moreover, organizing regular professional training, experience exchange and sharing activities and mentorship programs can offer practical assistance for the professional development of young teachers.

Another aspect the university have to pay attention to is the improvement of young university teachers job satisfaction. As we revealed, job satisfaction can not only directly effect young university teachers job burnout, but mediate the relationship between ERG need satisfaction and job burnout. Therefore, it is important to improve young university teachers job satisfaction to reduce burnout. In addition to the aforementioned need satisfaction, university can also improve teacher job satisfaction by enhancing teachers’ professional identity. Young university teachers are in a critical period for the formation of professional identity, which is an important intrinsic factor that affects teachers’ professional growth and has a positive impact on their job satisfaction (Lu et al., 2022).

## 6. Contributions and limitations

This study primarily contributes in the following aspects: First, using structural equation modeling, we revealed the relationship between ERG needs satisfaction, job satisfaction, and job burnout, thereby enriching the knowledge about the antecedents of young university teachers’ job burnout. Second, the path difference analysis demonstrated that the total effects of relatedness need satisfaction and growth need satisfaction on job burnout were significantly stronger than the total effect of relatedness need satisfaction among young university teachers, laying the groundwork for more in-depth identification of the mechanisms underlying the effects of various needs. Additionally, the findings of this study somewhat support the value of applying ERG theory to the group of young teachers.

However, there are still some limitations on this study. First, because to data constraints, we were unable to control for the country factors. However, given that there may be differences in young teachers’ working conditions between nations, it is important to proceed with caution when applying the findings. Future research could conduct a larger-scale survey in various countries to further explain whether this mechanism differs in different nations. Seccond, since the data used in this study are cross-sectional, we were unable to draw any causal inference about the relationship between ERG needs satisfaction and job burnout. Future studies could obtain survey data from the same sample group at different time points and then use longitudinal data analysis to investigate the causal connection between these two variables. Third, we only analyzed the relationship between ERG needs satisfaction and job burnout among young university teachers, so caution should be taken when generalizing the findings to university teachers of different age groups. Future research can increase comparisons across different age stages to improve its generalizability.

## Data availability statement

The original contributions presented in the study are included in the article/Supplementary material, further inquiries can be directed to the corresponding author.

## Ethics statement

Ethical review and approval was not required for the study on human participants in accordance with the local legislation and institutional requirements. Written informed consent for participation was not required for this study in accordance with the national legislation and the institutional requirements.

## Author contributions

YY led the project conception, conceptualization, theoretical direction, interpretation of the statistical analyzes and results, and original draft preparation. QL critically revised the manuscript and actively participated in finalizing the manuscript. All authors contributed to the article and approved the submitted version.

## Funding

This work was supported by the Fieldwork Funds for Graduate Students of Xiamen University (2022FG031).

## Conflict of interest

The authors declare that the research was conducted in the absence of any commercial or financial relationships that could be construed as a potential conflict of interest.

## Publisher’s note

All claims expressed in this article are solely those of the authors and do not necessarily represent those of their affiliated organizations, or those of the publisher, the editors and the reviewers. Any product that may be evaluated in this article, or claim that may be made by its manufacturer, is not guaranteed or endorsed by the publisher.
